# Animal welfare and effects of per-female stress on male and cattle reproduction—A review

**DOI:** 10.3389/fvets.2023.1083469

**Published:** 2023-03-31

**Authors:** Welligton Conceição da Silva, Jamile Andréa Rodrigues da Silva, Raimundo Nonato Colares Camargo-Júnior, Éder Bruno Rebelo da Silva, Maria Roseane Pereira dos Santos, Rinaldo Batista Viana, André Guimarães Maciel e Silva, Cleidson Manoel Gomes da Silva, José de Brito Lourenço-Júnior

**Affiliations:** ^1^Postgraduate Program in Animal Science (PPGCAN), Institute of Veterinary Medicine, Federal University of Para (UFPA), UFRA, Brazilian Agricultural Research Corporation (EMBRAPA), Castanhal, Brazil; ^2^Federal Rural University of the Amazon (UFRA), Institute of Animal Health and Production, Belém, Brazil; ^3^Federal Rural University of the Amazon (UFRA), Institute of Agricultural Sciences, Capanema, Brazil; ^4^Federal University of Western Pará (UFOPA), Institute of Engineering and Geosciences, Santarém, Brazil; ^5^Federal University of the South and Southeast of Pará (UNIFESSPA), Institute of Veterinary Medicine, Xinguara, Pará, Brazil

**Keywords:** heat load, thermal stress, reproduction, thermotolerance, spermatogenesis

## Abstract

Thermal stress causes severe effects on the wellbeing and reproduction of cattle, including changes in oogenesis and spermatogenesis, generating great concerns, which last for decades. In cattle, the occurrence of thermal stress is associated with a reduction in the production of spermatozoids and ovarian follicles, in addition to the increase of major and minor defects in gametes or in their intermediate stages. In bovine females able to reproduce, a reduction in the rate of estrus manifestation and an increase in embryonic mortality has been observed. Therefore, keeping animals on good welfare conditions, with water supply and in shaded areas can favor the improvement of different reproductive parameters. For all this, the present study aimed to gather, synthesize and argue recent studies related to animal welfare, focusing on the effects of thermal stress on the reproduction of cattle, aiming to support possible strategies to mitigate the harmful effects of thermal stress in this species.

## Introduction

Animal welfare (AW) is defined as the physical or mental state of an animal in relation to the environment in which it lives and dies (1). Thus, a good degree of AW means to say that an individual is safe, healthy, comfortable, well-nourished and free to express natural behaviors of the species without suffering from harmful mental states such as pain, frustration and stress.

The behavioral reactions observed in animals can be determined by the way they react to the environment, with animals of other species or the same, as well as humans, which can directly interfere with the change in their behavior, posture and attitude (2). When the animals have behavioral characteristics defined as normal in an unknown environment, it is believed that they have a good degree of AW, for example, the practice of rumination and the waiting period signals tranquility (3).

According to the five domains of animal welfare proposed by Mellor et al. (4), the environment (for example, physical and atmospheric characteristics) impacts the physical and mental health of all species.

In cattle, one of the most mentioned causes capable of reducing their well-being are thermal conditions, that is: temperatures that are too high or too low, due to anatomical reasons or the places where they are raised (5).

Several consequences derived from heat stress can be cited, such as reduced oocyte and sperm quality. Thus, in tropical climate regions these variables express greater influence on the thermal comfort of cattle, a fact that worsens even more in pastures without the presence of trees or shading, because the animals start to receive high solar radiation, and are exposed to critical temperatures and high relative humidity, resulting in thermal stress (4).

Most livestock species, including cattle, define their thermal comfort zone between 16 and 25°C, that is, in this range of ambient temperature the basal metabolism is lower and therefore thermoregulation takes place without evapotranspiration (6).

Several studies have been carried out to measure the effects resulting from microclimates that interfere with the thermal comfort of cattle, the main variables being measurable; temperature, humidity, wind speed and solar radiation that are used to determine the indices of thermal comfort in animals (7–13).

**Figure 1:**
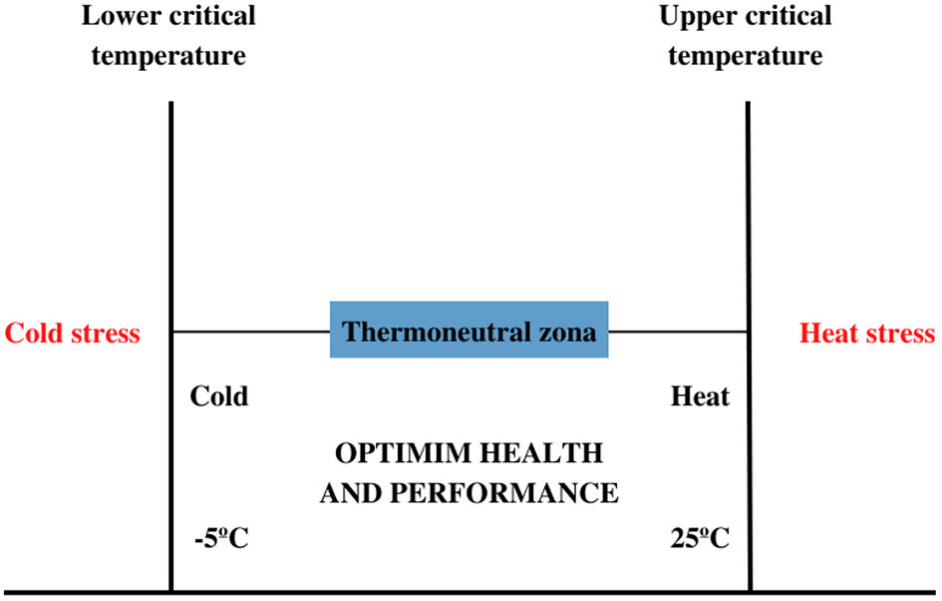
Critical temperatures and thermo-neutral zone in dairy cattle. Adapted from Avendaño-Reyes (15).

Cattle, being a homeothermic animal, have the ability to control body temperature when exposed to large temperature variations, and the thermoregulation mechanism is responsible for this balance (14). Thus, when the animal is exposed to critical temperatures, sweating, increased water intake, respiratory rate and vasodilation can occur, and when the ambient temperature is higher than the thermoneutrality zone (Figure 1) thermal stress can occur, harming wellbeing, health and reproduction (16).

For all these reasons, the main objective of this research was to analyze scientific information on welfare, but also to gather, synthesize and discuss recent discoveries, with a focus on heat stress in bovine reproduction, providing information capable of supporting strategies to mitigate its negative effects in this species.

## Generalities about animal welfare

The AW theme is widely discussed in animal production. Historically, it began in the United Kingdom in 1964, with different reports published by journalist Ruth Harrison, warning of the need to observe animals as living beings and not mechanically, as inert beings (17).

In 1965, the British Parliament set up a committee coordinated by Professor Rogers Brambell to provide information to livestock farmers on how to raise animals. The guidelines set out in the report were known as: “Brambell's five freedoms”, such as: “turn around”, “lie down”, “get up”, “scan your limbs” and “take care of your own body” (18). After these publications, different views were established about the AW, seeking to create criteria in order to measure this parameter in the animals.

In conceptual terms, the definition of the most accepted AW was established by Broom (19), who described it as the state of an individual during his attempts to balance himself in an environment. Thus, when animals are placed in different environments or subjected to inadequate management, they tend to present stress, which results in negative aspects in production (20).

**Figure 2:**
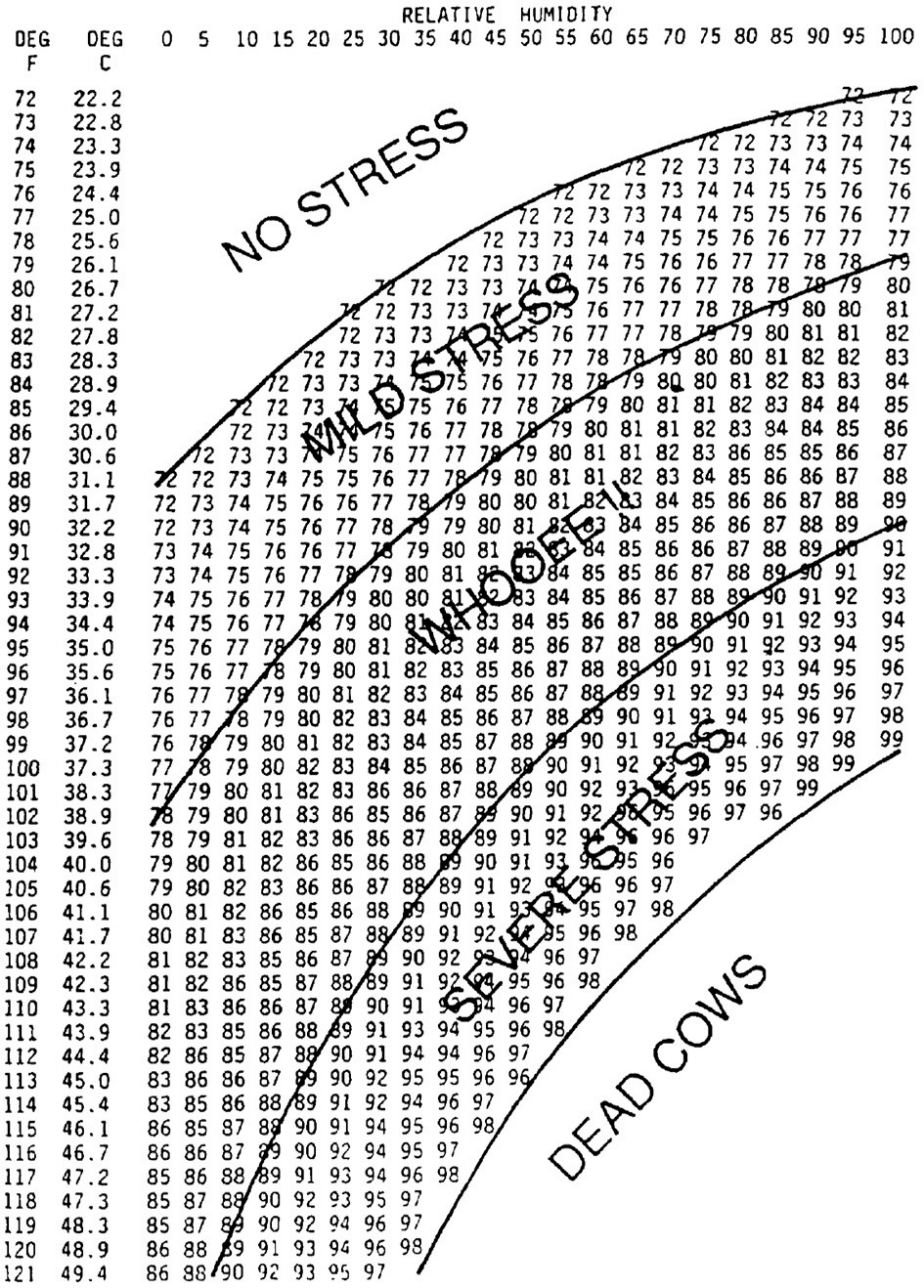
Temperature-humidity index (THI) table for dairy producer to estimate heat stress for dairy cows. Deg, Degrees. Relative humidity expressed as percentage. Adapted from Armstrong (23).

The five freedoms are presented as an important milestone in the science of AW, where the animal must be: 1. free from hunger and thirst, with free access to water and diet to maintain health and vigor; 2. free from discomfort, with an appropriate environment, with shelter and comfortable waiting area; 3. free from pain, injury and illness, with rapid prevention, diagnosis and treatment; 4. free to express normal and natural behavior, with sufficient space, adequate and species-specific facilities; and 5. freedom from fear and distress, with conditions and treatment that prevent mental suffering (21). However, it is worth noting that the FAWC suggests that the five freedoms should not be adopted as minimum animal welfare standards (22) (Figure 2).

In 2004, the European Union presented the “Welfare Quality^®^ Project”, stimulating integration between researchers about AW from different institutions. Four approaches and 12 criteria were proposed (23), such as: Good nutrition, defined by absence of hunger and prolonged thirst (1 and 2); Good accommodation, for waiting (3) and providing thermal comfort (4), in addition to ease of movement (5); Good health, characterized by the absence of injuries (24), diseases (6), pain induced by handling (7); and present appropriate behavior, such as: the expression of social behavior (8), of other behaviors (9), in addition to having a good interaction between human and animal (10) and positive emotional states (11).

Mellor (25) proposed the model of the five domains, seeking to encompass the negative and positive aspects related to animals, divided into four physical principles and one mental, namely: nutrition, environment, health, behavior and the mental state of animals, considering- whether positive and negative emotions and feelings are presented. Thus, the search for positive AW indicators is essential, combined with measures that aim to maximize the expression of normal behaviors (26).

## Key animal welfare indicators

To understand AW, it is necessary to know the species to be studied, considering the behavioral characteristics in the natural habitat, as well as the breeding system of the individuals. When trying to measure AW, attention should be paid to a variety of multidimensional indicators, in addition to different parameters to understand the general state of everyone (27).

AW indicators can be classified into two types: the first is related to animals that cannot adapt to a certain environment, the second represents the effort made by the animal to adapt to the environment (28).

Mismanagement errors during the animal's adaptation process in a given environment can cause problems such as an increase in the mortality rate (29) (Table 1) and a decrease in live-born calves (30). Attempts to adapt to the new environment may present different indicators, such as an increase in the number of diseases (31), a decrease in milk production (32) (Table 2) and in the growth rate of animals (34).

**Table 1:** Neonatal mortality rates in different farm animals in different countries.

**Animal**	**Overall mortality rate (%)**	**Climate (country)—source**
Lambs	10–25	Temperate to cold-temperate (Australia, NZ, UK)
Kids	15–51	Temperate/tropical (India)
	12–50	Temperate (South Africa)
	7–17	Warm-temperate (Mexico)
	15–50	Cold-temperate (NZ)
Bovine calves	8	Indoors (Canada)
	50	Outdoors (Canada)
	6–15^a^	USA
	0–10	UK
Deer calves	10–12	Farmed (NZ)
	10–90^b^	Natural herds (USA)
Foals	0–35^b^	Natural herd (USA)
	30^c^	Natural herd (NZ)
Piglets	5–20	Indoors (UK)
	12–19^b^	With farrowing crate
	13–35^b^	Without farrowing crate

NZ, New Zealand; UK, United Kingdom; and USA, United States of America.

a Mortality in first 24 h.

b Preweaning mortality rates.

c Loss from pregnancy to newborn foals at foot. Adapted from Mellor and Stafford (29).

**Table 2:** Effect of heat stress on dairy cattle.

**THI**	**Stress level**	**Effects**
22.2°C (< 72°F)	None
22.2–26.1°C (72–79°F)	Mild	Dairy cows will adjust by seeking shade, increasing respiration rate and dilation of the blood vessels. The effect on milk production will be minimal.
26.6–31.6°C (80–89°F)	Moderate	Both saliva production and respiration rate will increase. Feed intake may be depressed and water consumption will increase. There will be an increase in body temperature. Milk production and reproduction will be decreased.
32.2–36.6°C (90–98°F)	Severe	Cows will become very much uncomfortable due to high body temperature, rapid respiration (panting) and excessive saliva production. Milk production and reproduction will be markedly decreased.
>36.6°C (>98°F)	Danger	Potential cow deaths can occur.

THI, temperature humidity index. Adapted from Samal (33).

**Figure 3:**
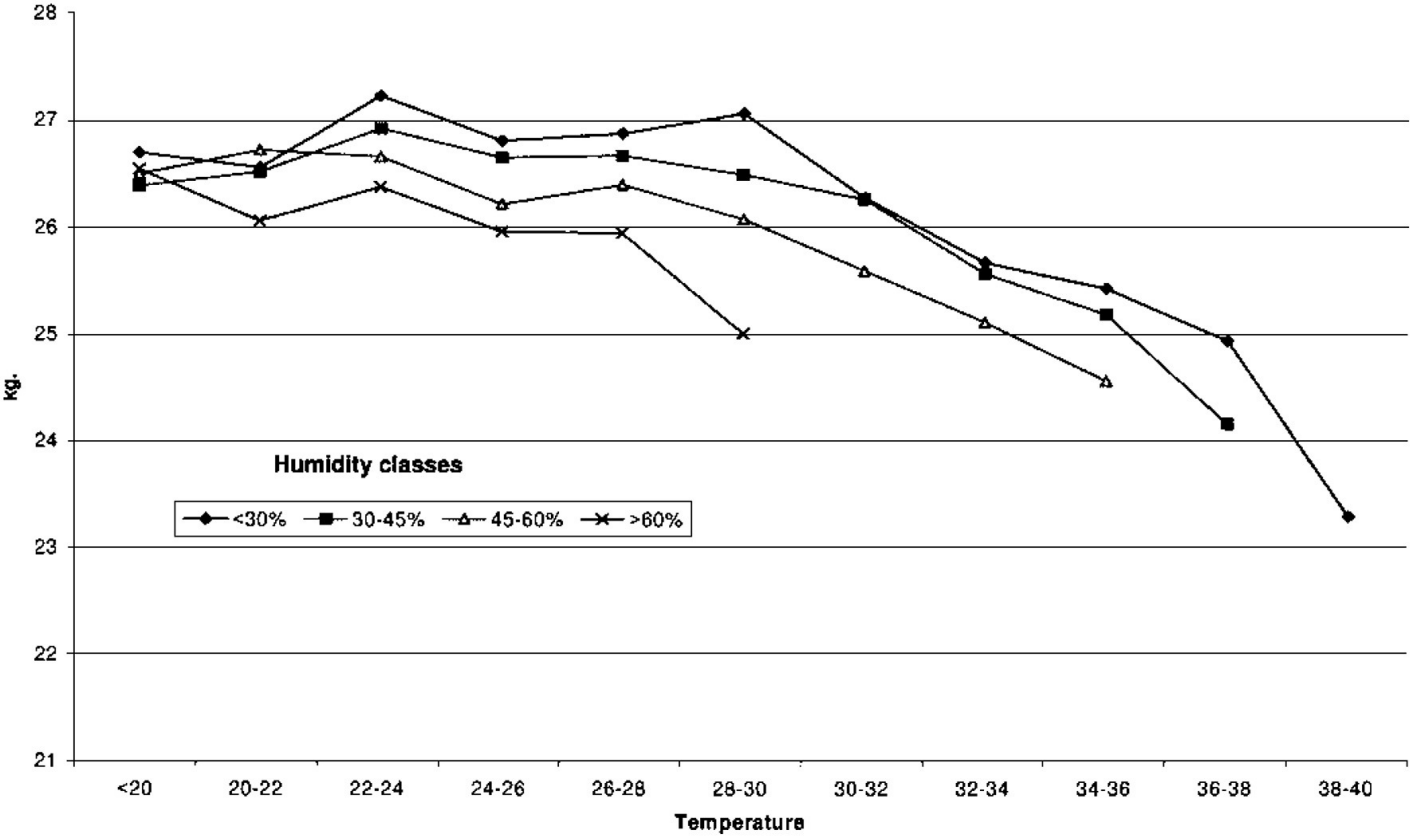
For milk production, the yield was relatively constant up to about 24°C and then began to decline as the temperature increased. Adapted from the study developed by Ravagnolo et al. (35).

Another important factor is the milk production index, which is also affected when heat stress is evident in dairy cattle (Figure 3).

**Figure 4:**
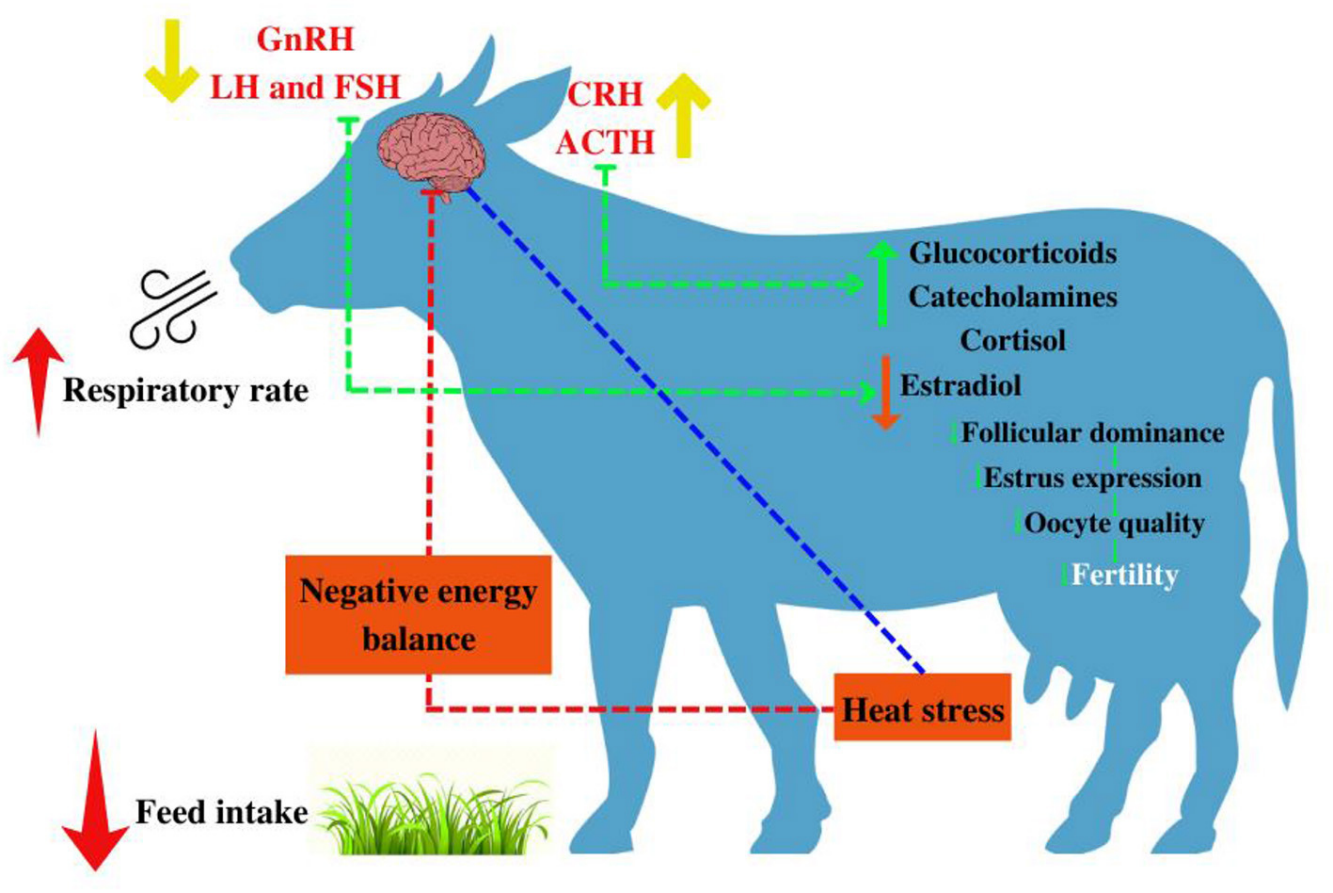
Impact of heat stress on female reproductive performance. Adapted from Krishnan et al. (37). Mitigation of the heat stress impact in livestock reproduction.

**Figure 5:**
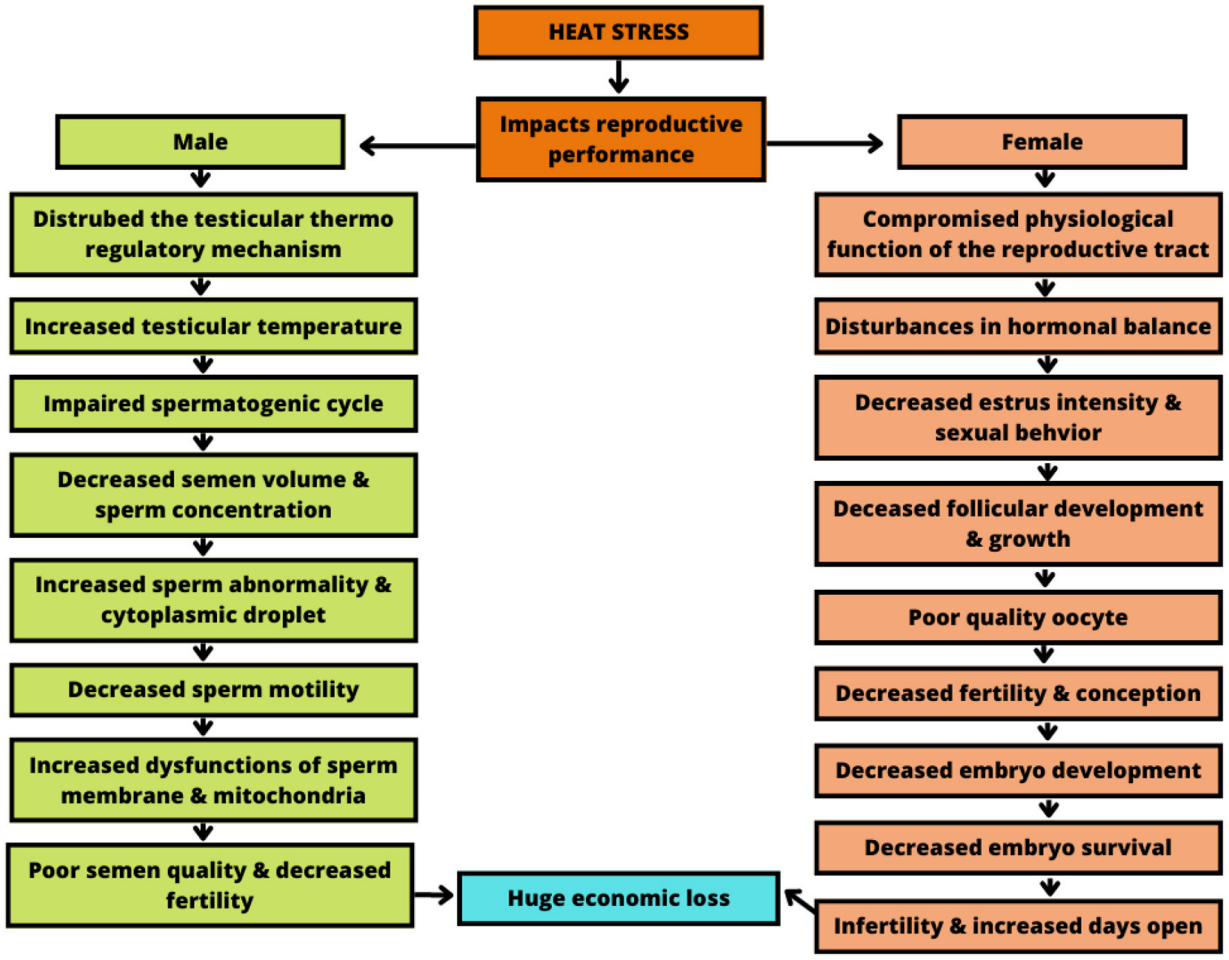
Impact of heat stress on pregnancy in livestock. Adapted from Krishnan et al. (37). Mitigation of the heat stress impact in livestock reproduction.

**Figure 6:**
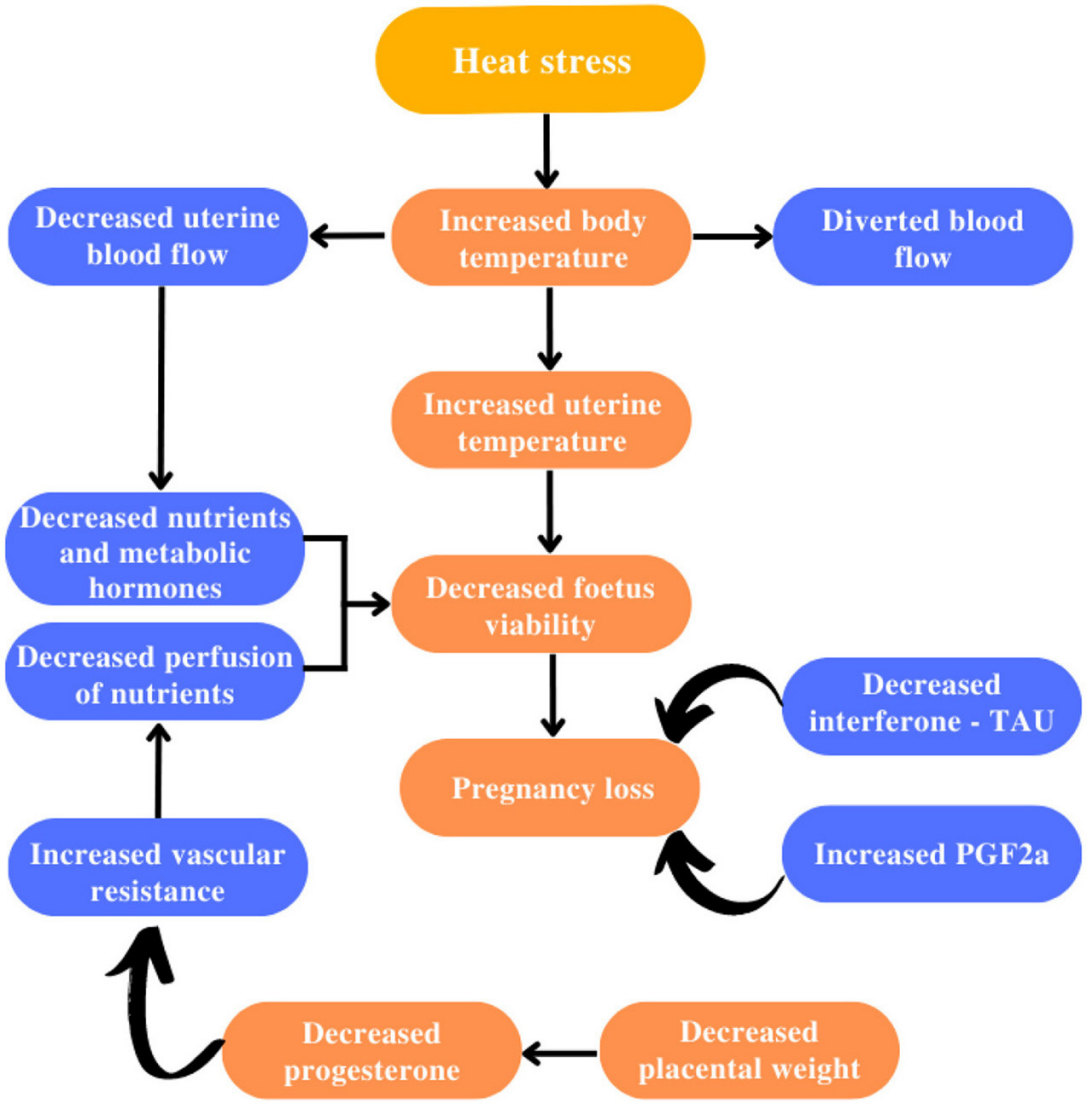
Aspects of the main impacts caused by heat stress during cattle handling. Adapted from Krishnan et al. (37).

Regarding fertility (36), heat stress promotes deleterious effects at all stages (Figures 4–6). Starting from the development of the oocyte (38, 39), continuing through the later stages, as well as its fertilization capacity (40). It is also harmful to the estrous cycle and estrous behavior (41). As well as the development and implantation of the embryo, persisting in the uterine environment, extending to the fetal calf (42).

The assessment of the degree of AW can be performed under different conditions, such as short-term conditions, for example, in the case of pre-slaughter management, instantaneous indicators such as heart rate, blood cortisol levels and the different behaviors of the animals during the slaughter are used (43), or conditions of prolonged duration, occurring in rearing systems, in which animals have a longer time to adapt (44).

AW is associated with different indicators that arise in response to forms of adaptation of environments, evidenced among animals; therefore, the adoption of a single indicator is an unfeasible alternative (28). In this sense, Botreau et al. (45) provided indicators that can be adopted in the evaluation of AW, such as the principle of good food, good accommodation, good health and expression of appropriate behavior.

In the first principle “good nutrition”, two criteria were defined that address issues related to the absence of hunger and thirst for long periods. In the second, described as “good accommodation”, comfort criteria were formulated related to the animals' waiting place, thermal comfort and mobility within the facilities. The third, “good health”, follows the criteria of absence of injuries, diseases and pain caused during the handling of the animals. Finally, the fourth criterion: “appropriate behavior”, has as its principle the expression of adequate social behavior and other behaviors, a harmonious relationship between the human being and the animal and the positive emotional state of the animals (45, 46).

Thus, in each criterion, we sought to identify specific measures that can be adopted to carry out the AW assessment, giving importance to its validity, repeatability and feasibility (47). It is worth mentioning that the measures established aim to evaluate the welfare of the animals individually, as it allows acquiring information directly linked to the adaptation of the animal to the environment, pointing out its performance in the breeding system, during a given production cycle.

Four principles are intrinsically linked to the reproduction of cattle and can be used when evaluating the AW, they are: nutrition related to the supply of water, food and essential nutrients; the environment, which represents the environmental challenges that are inserted; health linked to diseases, injuries and functional impairment; animal behavior, and, finally, the mental state of animals, which considers the positive and negative emotions and feelings presented (48).

## Behavioral indicators of AW in cattle

The behavioral reactions observed in animals can be determined by the way they react to the environment, animals of other species or the same, as well as the presence of human beings, which can directly interfere in the change of their behavior, attitude and posture (2). Communication between cattle is carried out through their senses and their behaviors are linked to their perception ability to relate sensory functions such as vision, hearing, smell and touch (49).

Bovines use visual signals as a strong means of communication, because due to issues related to the evolutionary process, these animals have anatomically large and well-developed eyes, in addition to panoramic vision (320°), which help in their survival. Visual sign language or body movements may relate to body movement or just a part of it (50).

Regarding body movement, the head and tail deserve to be highlighted. They have great mobility and allow different movements in relation to the body, being able to express different types of information, mainly during aggressive or submissive behavior (2), constituting, therefore, a strong mood indicator (51).

**Figure 7:**
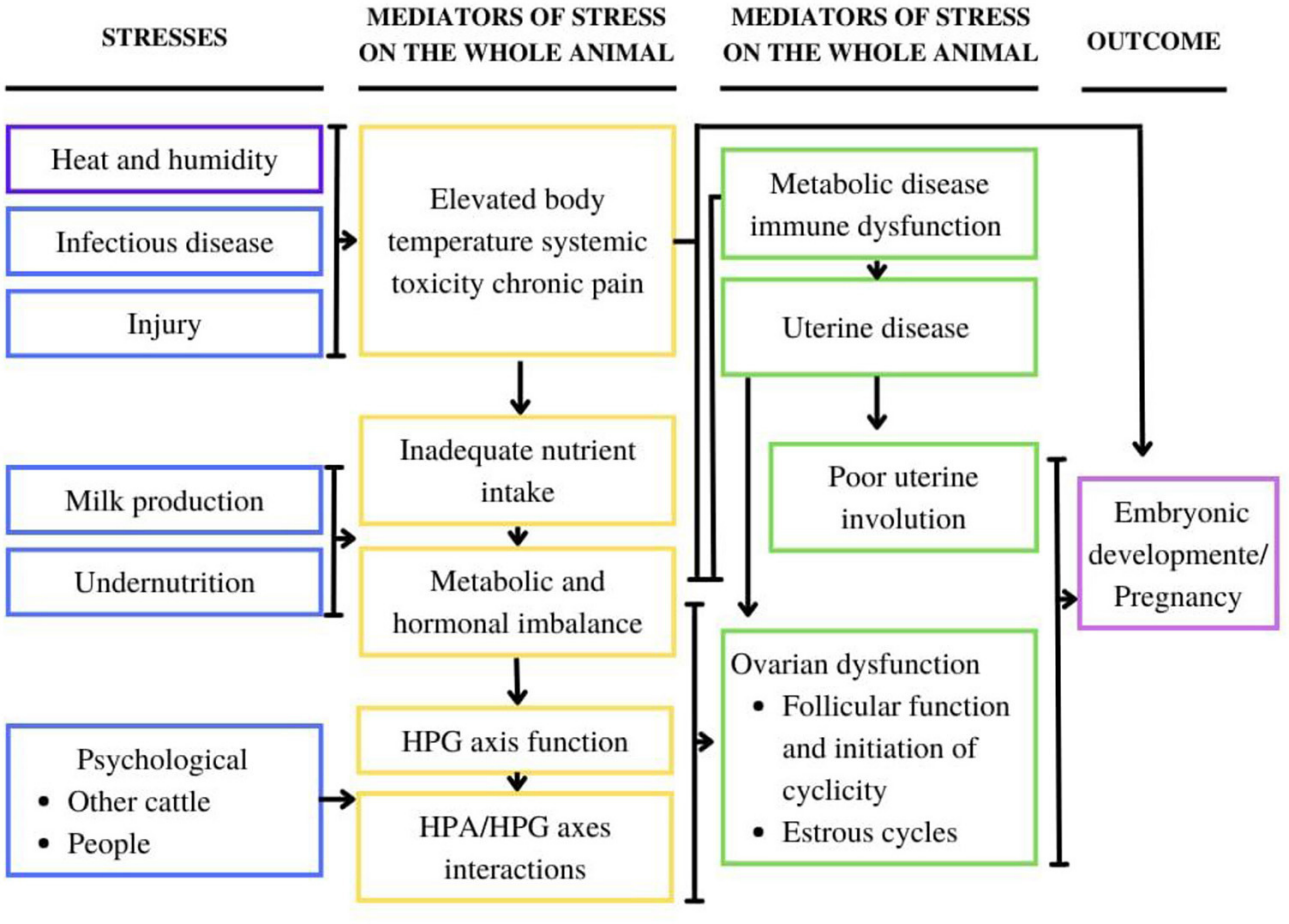
Schematic drawing on the main aspects that affect cattle, as well as the mediators of stress in the animal, and finally the associated strain that affects the pregnancy outcome. HPG, hypothalamic-pituitary-gonadal; HPA, hypothalamic-pituitary-adrenal. Adapted from Matthew (52).

The vocalizations of cattle do not prove to be specific to a given situation, but mainly due to the degree of arousal, being reported mainly during situations of stress (Figure 7, Table 3), frustration and pain (50, 53).

**Table 3:** Sources of stress and recent review papers written on the topic.

**Sources of stress**
Infectious disease of the reproductive tract
Injury
Heat
Metabolic imbalance postpartum
Social/psychological
Nutritional
Transportation

Adapted from Matthew (52).

The following parameters are recommended for interpreting vocalization levels: if 1% of the cattle emit sound (excellent), 3% (acceptable), 4–10% (not acceptable) and >10% indicate serious problems related to welfare (54). When an animal continuously and intensely avoids a situation, it externalizes information related to its degree of wellbeing. Thus, an accentuated avoidance reaction, during the presence of a stimulus, can signal a good or bad degree of welfare (55).

When an animal cannot express a natural behavior, even trying to perform it at different times, it has a poor degree of wellbeing when compared to another animal that can perform the behavior. For this reason, knowing the behavior of animals in a natural environment makes it more feasible to evaluate the behavior of individuals in a different condition than the natural one, allowing a more accurate diagnosis of welfare (56).

## Stress

Stress can be defined as the set of physiological reactions that occurs in an organism in order to adapt to different situations. However, these reactions can cause imbalance, depending on the intensity and duration (57). The emotional state of the animal, whether positive or negative, can cause stress. However, the adaptation to the new environment will provide the restoration of balance or return to the normal state (58).

The animal organism tends to prioritize homeostasis, however, when subjected to factors that trigger stress, they may respond through a combination of biochemical, physiological and behavioral reactions (2).

The General Adaptation Syndrome (GAS) is divided into three different phases: the first phase, also called alarm or alert, is characterized by the response of the Sympathetic Nervous System (SNS), which signals the activation of the adrenal glands, with secretion of hormones cortisol, adrenaline and noradrenaline. As a consequence, there will be tachycardia, tachypnea and elevated blood glucose levels (59).

**Figure 8:**
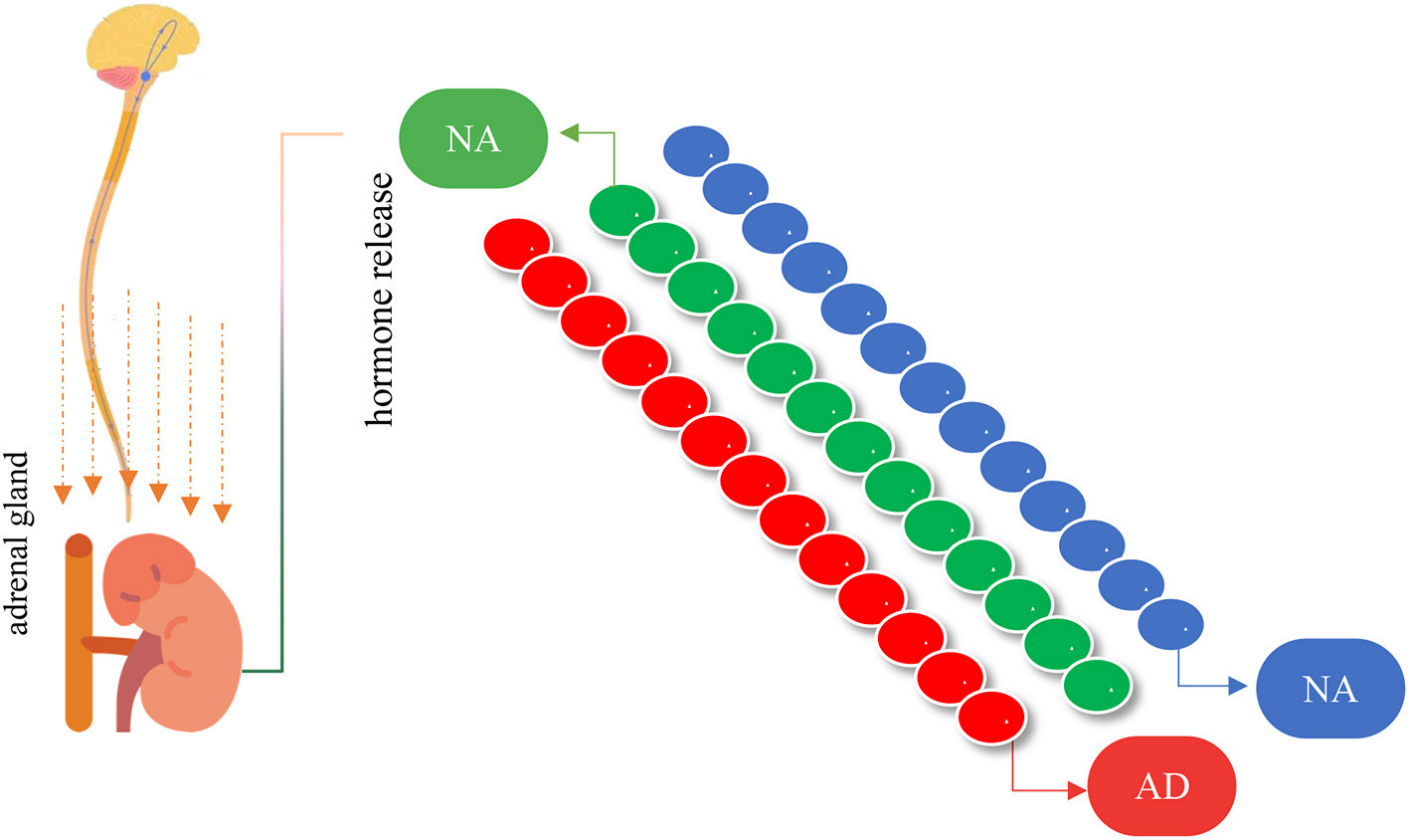
Illustration of the initial response when there is a stressor stimulus in cattle. The sympathetic nervous system stimulates the adrenal gland to release catecholamines [adrenaline (AD) and noradrenaline (NAD)] and cortisol (CT).

The second phase (adaptation or resistance) is characterized by the continuous secretion of glucocorticoid hormones (Figure 8), providing the animal's organism with a considerable improvement in physical and cognitive activity, which aims to nullify the aggressor (59). Finally, the last and third phase begins, called exhaustion, when the animals are subjected to intense and prolonged stress, which thus preserves the body's response. Thus, stress becomes chronic, which promotes physiological reactions and changes in behavior, in addition to causing energy overload and system exhaustion (60).

## Heat stress

The sum of external environmental forces acting on animals is defined as heat stress, which results in an increase in body temperature and causes a physiological response (61). Reduced productivity, decreased wellbeing, increased susceptibility to diseases and decreased fertility are effects of heat stress, which in extreme situations increase the mortality rate (62) and cause negative effects on all the domesticated species (63).

Thus, among the parameters affected by heat stress, the energy reserves of cattle stand out (64, 65), impairing the development of beef cattle and lactating cows, as these animals need good nutrition. Heat dissipation is the animal's main response to heat stress (64) which takes place through behavioral and physiological thermoregulatory mechanisms (65–67).

In this scenario, these mechanisms dissipate heat through sweating and peripheral circulation, increased respiration, wheezing, and decreased rate of food intake to retain metabolic heat (67). Finally, the detailed mechanism of heat dissipation in cattle exposed to heat stress was be reviewed by Berman (68) and Collier et al. (69).

**Figure 9:**
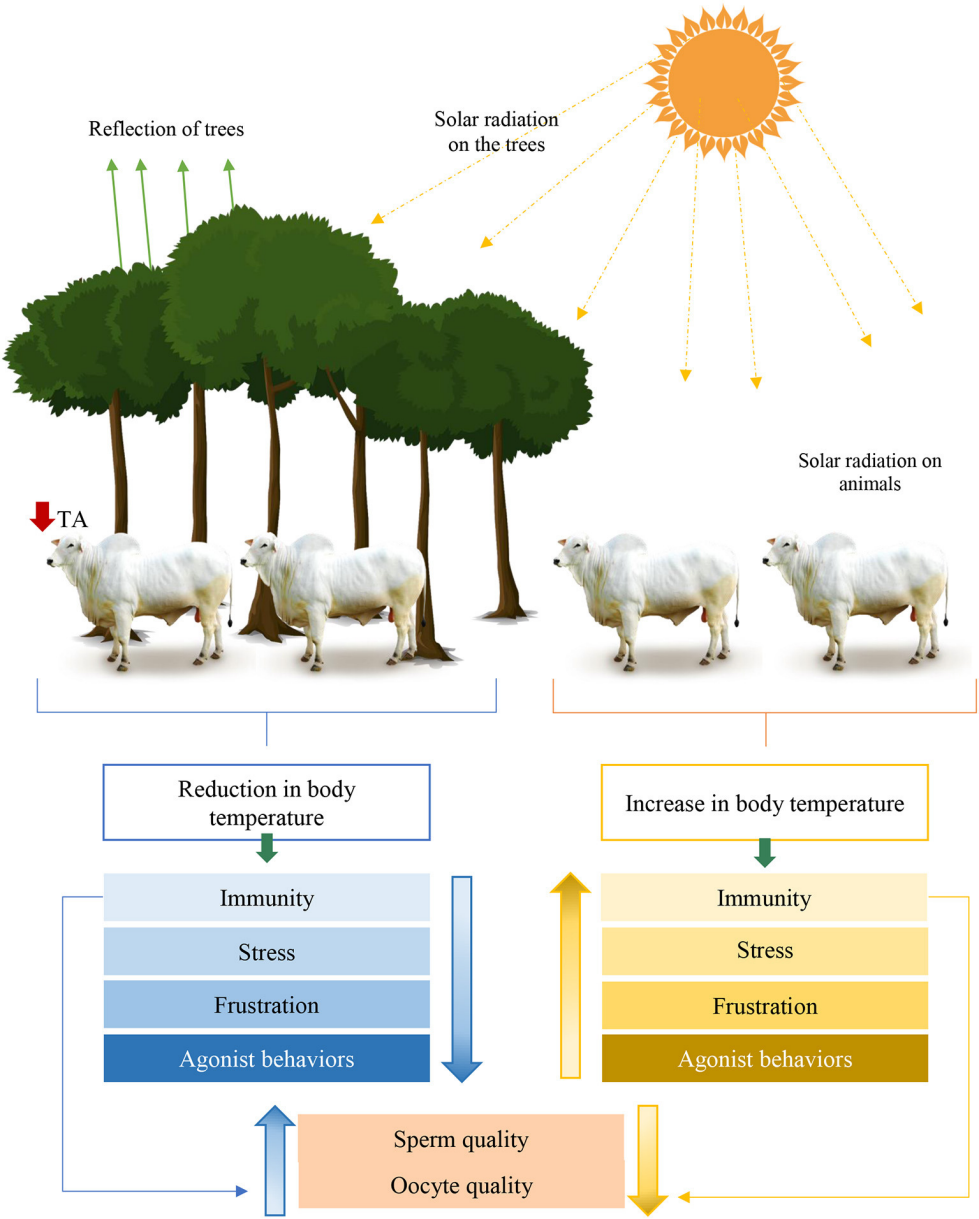
Infographic of environmental effects on physiological, behavioral and stress parameters in male and female cattle conditioned in sun and shade systems. Adapted from Edwards-Callaway et al. (82). TA, Ambient Temperature.

Other studies sought to investigate the consequences caused by heat stress on the physiology of dairy cattle [for example, (70–73)] and confined livestock [for example, (74–81)] with revision limitation in the cow-calf sector. Note that most mechanisms are consistent between beef and dairy cattle. The different visual mechanisms of heat stress in beef cattle can be seen in Figure 9.

## Egg loss and early embryo loss caused by heat stress

Embryonic death is considered one of the main causes of low reproductive efficiency in cattle, reaching 25–40% of cases and can be divided into early and late, and early can record 40% of embryonic losses. In bovids, the occurrence of early embryonic losses begins between the 7th and 16th day of gestation, during the hatching of the blastocyst and its subsequent implantation, without interfering with the extension of the cycles (83).

In addition, it directly harms production and economic investment in the sector. For this reason, some parameters seek to be addressed in order to reduce cases of embryonic loss and ensure pregnancy rates in cattle. Regarding the clinical factors manifested by early embryonic loss, progesterone deficiency, inbreeding, multiple pregnancy, incompatibility, chromosomal aberrations and heat stress, among others, stand out.

**Figure 10:**
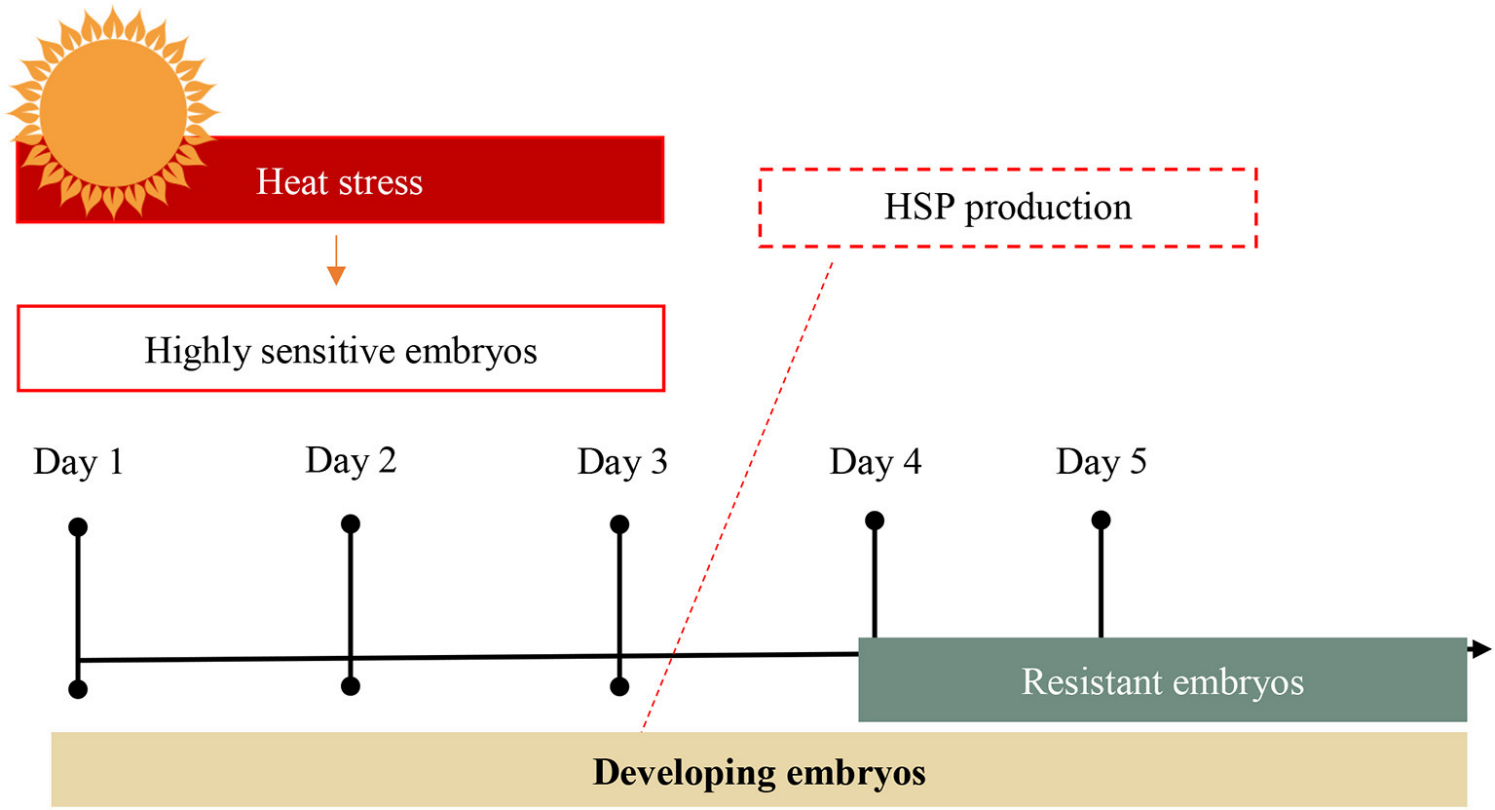
Effects of heat stress on bovine embryo development and heat shock protein (HSP) production (42).

Heat stress is related to early embryonic losses, more common in high dairy animals. Due to the presence of greater productive capacity, there is a greater intake of dry matter, requiring an instantaneous metabolic work, causing the animal to produce more heat. Heat stress between the 1st and 3rd days of embryo development decreases embryo viability due to its high sensitivity (42) (Figure 10).

In dairy cows subjected to heat stress, there is a tendency to present fertility problems. This is due to different factors, affecting from the manifestations of estrus signs, to changes in follicular function, ovulation, or even promoting embryonic death (Table 4) (72, 84, 99–103).

**Table 4:** Reproductive problems identified in dairy cows under heat stress.

**Problems identified**	**References**
Lower estrus detection rates.	Collier et al. (84)
Reduction in the size of the ovarian follicle.	Wilson et al. (85), Schüller et al. (86)
Altered follicular fluid composition combined with abnormal concentrations of ovarian steroids.	Alves et al. (87), Roth and Wolfenson (88)
Relative location, morphology, and function of ooplasmic organelles (especially mitochondria) are altered by heat stress, and these changes are especially apparent in oocytes of *Bos taurus taurus* origin.	Silva et al. (89), Maya-Soriano et al. (90)
Transient effects up to 50 days after exposure to heat stress.	Lussier et al. (91), Roth et al. (92)
Decreased sperm concentration and motility and increased morphological abnormalities.	Malama et al. (93), Sabés-Alsina et al. (94), Rahman et al. (95)
Abnormal condensation of chromatin in sperm.	Rahman et al. (95), Lúcio et al. (96)
Early embryonic death.	Ealy et al. (42), Edwards and Hansen (97)

Source: Adapted from Negrón-Pérez et al. (98).

## Effects of heat stress on female reproduction

Heat stress is defined as one of the limiting and essential factors in the dairy production sector, even though the bovine property is maintained under controlled environmental techniques. Severe heat waves in early summer can be harmful to the animal when it is not in a climate-controlled environment (104, 105). For this reason, heat stress in bovine reproduction has been widely addressed in recent decades (52, 106–114).

In this context, it is observed that heat stress has had negative consequences in terms of reproductive function. Among these effects, oocyte maturation, early embryonic development, fetal and placental growth and lactation stand out (115). It is noteworthy that the unfavorable effects are related to the situation of hyperthermia or physiological changes developed by the animal in a situation of thermal stress, making it impossible for this individual to dissipate heat to regulate body temperature (105).

The impacts of heat stress on reproductive function affect the formation and function of male and female gametes, embryonic development, as well as fetal growth and development. The proportion of these effects is greater in high producing cows than in low producing cows and non-lactating heifers. The result of this process can occur immediately or late in various reproductive tissues and reproduction processes (101, 115–118).

## Effects on ovarian follicle development and dynamics

The most evident effect of heat stress that interferes with reproduction occurs through deleterious mechanisms on ovarian follicles. Heat stress tends to alter follicular growth and development in addition to affecting ovarian follicular dynamics during the estrous cycle (119–121). Follicles developed exposed to temperatures close to 40°C can be permanently damaged and become unviable (85). The identification of these damages can be expressed after a short interval or belatedly (122, 123).

Some authors emphasize that heat stress delays follicle selection and, to make matters worse, prolongs the interval of the follicular wave and follicular steroidogenesis. All of this results in suppression of large follicles, as the dominant follicle fails to exert dominance (120, 124, 125).

The effects can be evidenced through the incidence of large follicles and follicular codominance. However, there are no reports of the immediate effect of heat stress on the small, medium and large follicles present in Girolando cows (123). The period of follicular growth can occur before the antral phase (42 days), or in the primary follicle (85 days), or in the period above three estrous cycles (123).

Heat stress interferes with follicular growth and dominance in the pre-ovulatory phase. In heat-stressed cows and heifers, dominant and subordinate follicles are smaller and larger, respectively, resulting in codominance (126–129).

It is noteworthy that the follicular proportion of the first wave is similar, however the secondary wave in thermoneutral cows has a larger diameter when compared to animals under heat stress (120). On the other hand, different results were obtained in Holstein cows, in which heat stress had a permanent negative effect on the antral follicles, especially in the period between 40 and 50 days after growth (130).

## Effect of heat stress on oocyte development

Oocytes are temperature-sensitive female germ cells that are subject to heat shock (131–133), remaining vulnerable to heat stress during the pre-ovulatory period (101). According to Paula Lopes et al. (133), the germinal vesicle and maturing oocytes are among the main ones affected by the adverse effects of heat stress. In this way, cows affected by heat stress have the quality of the gamete impaired, since the stressed animal cannot obtain greater production of oocytes.

Heat stress causes cellular damage to the cytoplasm and nucleus of the oocyte. In addition, it can stimulate apoptosis, compromise cytoskeleton and mitochondrial function. All this will end up harming the quality of oocyte development (132, 133).

The cytoplasm is the part most susceptible to heat shock compared to the nucleus (133), this anomaly is closely linked to changes in the amount of progesterone, LH and FSH secreted during the estrous cycle (105, 122, 134).

The reaction to heat stress in *Bos taurus taurus* and *Bos taurus indicus* cattle shows negative and late results regarding oocyte competence (123). Thus, the development of small follicles can be impaired by heat stress during the summer and lead to ovulation of immature oocytes (135).

## Influence of heat stress on fertilization, embryonic development and early embryonic losses

For Hansen et al. (115), heat stress impairs oocyte quality and fertilization rate, which can result in the formation of poor quality embryos (116, 131, 132).

According to Wolfenson et al. (117), the early embryo is the most susceptible to the effects of heat stress. Thus, the deleterious effect becomes more worrying when it occurs in the first divisions of cleavage, or when part of the embryonic genome is inactive. After that, depending on the progress of its development, the bovine embryo gradually acquires greater resistance to the increase in temperature (105). Paula Lopes et al. (133) emphasize that the consequences of heat stress are less common in heat-tolerant breeds (*Bos taurus indicus*) when comparing them with heat-sensitive or exotic breeds.

Cows under heat stress tend to lose their embryos before completing 42 days (101, 136, 137). This can be explained by the fact that in less heat-tolerant breeds, there is a decrease in blood flow to the ovaries, causing a reduction in the supply of nutrients and hormones, which will interfere unfavorably with embryonic development. This scenario can lead to increased production of PGF2y promoting premature luteolysis and early embryonic mortality (138).

## Heat stress in the male bovine

### Consequences of increased testicular temperature due to climate change

The process of transpiration and respiration in cattle has the function of dissipating the heat retained in their body, but these mechanisms are not so favorable to transfer heat to the environment, thus characterizing the limit of ambient temperature as thermal stress (139).

One of the mechanisms responsible for the occurrence of testicular heat stress is the increase in ambient temperature provided by global climate change (140). Given this condition, several studies have reported the negative effects of increased environmental temperatures on male reproduction (141).

It was observed in bulls exposed to high ambient temperatures (40°C), lower sperm quality, in other words, a reduction in the percentage of motile sperm and an increase in the percentage of abnormal sperm (142–144).

In addition, there is a significant reduction in testicular blood flow during the warmer months, causing deleterious effects on seminal plasma enzymatic activity and various aspects of semen quality (145).

In a study carried out with Simmental (*Bos taurus*) bulls, Koivisto et al. (146) reported higher rates of sperm defects in summer compared to winter, because younger animals are more sensitive to high ambient temperatures.

These studies help to clarify why bulls more resistant to heat tend to have a higher quality of thawed semen, varying throughout the year, due to the occurrence of a high proportion of ejaculates discarded during the summer (147, 148) (Table 5).

**Table 5:** Morphological defects observed in studies after heat stress due to scrotal isolation in cattle.

**Heat stroke period**	**Days on which sperm morphological abnormalities are evidenced in semen after heatstroke**	**Observed morphological defects**	**References**
48 h	6–23 days	Decapitated sperm, abnormal acrosomes, abnormal tails, and protoplasmic droplets	Wildeu and Entwhistle (149)
48 h	12–36 days	Tailless sperm, diadem defects, piriform head, nuclear vacuoles, knotty acrosomes, and drag defects	Vogler et al. (150, 151)
48 h	14–42 days	Piriform heads, large heads, nuclear vacuoles	Rahman et al. (152)
48 h	23–34 days	Piriform heads, vesicle formation in the equatorial region of the sperm head (diadem defect), apical vacuoles	Walters et al. (153)
72 h	15–49 days	Piriform heads, detached heads, midpiece defects, proximal droplets	Newton et al. (154)
96 h	18–25 days	Piriform heads, nuclear vacuoles, microcephalic sperm and abnormal DNA condensation	Barth and Bowman (155)
120 h	14–21 days	Head abnormalities, nuclear vacuoles, acrosome and midpiece defects	Fernandes et al. (156)

Source: Adapted from Rahman et al. (95).

## Effects of heat stress on spermatogenesis and semen quality

The direct effects of heat stress on sperm production can also impact sperm quality, through the proportion and duration of testicular heating. Sperm morphology can be altered by an increase in testicular temperature, although they may remain normal for a few days if the epididymal spermatozoa are minimally affected, followed gradually by the emergence of morphologically abnormal sperm (157).

In contrast, elevated testicular temperatures were observed in spermatozoa in the epididymis (150, 151, 158, 159), which after 2 days of scrotal isolation were adversely affected, showing reduced mobility and acrosomal integrity capacity.

As for sperm reproduction, Vogler et al. (151) report several morphological defects (Table 6) that are evidenced by the distal reflexes of the midpiece, by the proximal and distal droplets and by the knotty acrosomes that appeared when reaching the peak between 11 and 18 days post-isolation. The aforementioned team also reports the identification of microcephalic and teratoid spermatozoa, with nuclear vacuoles, pyriform heads, in addition to curled tails.

**Table 6:** Anomalies evidenced in sperm after heat stress in cattle.

**Anomalies**	**References**
The increase in GPx activity was insufficient to minimize the damage caused by the amount of ROS produced during the summer.	Nichi et al. (147)
Partial impairment of motility, genetic modifications and heritability	Al-Kanaan et al. (160)
Significant DNA fragmentation in summer	Valeanu et al. (161)
Decreased percentage of live sperm, reduced amount of sperm per unit volume of semen	Sharma et al. (162)
Decreased seminal pH and reduced plasma membrane	Sharma (163)
The occurrence of heat stress during spermatogenesis affects seminal quality	Sabés-Alcina et al. (164)
Increased defects in sperm morphology, sperm DNA fragmentation, elevation of lipid rate, mitochondrial membrane modification potential, sperm motility and IMP	Garcia-Oliveros et al. (165)
Semen collected in periods with high indices of Temperature and Humidity Index (THI) present reduced viability in vitro favoring the reduction of blastocysts and delay in hatching	Luceno et al. (166)
Commitment to viability and seminal quality	Residiwati et al. (167)
Nutritional factors associated with climatic conditions alter the concentrations of NPY e ATP1A1	Pires et al. (168)

IMP, plasma membrane integrity; NPY, neuropeptide Y; ATP1A1, ATPase Na+/K+ transporting subunit alpha 1; DNA, Deoxyribonucleic Acid; THI, Temperature and Humidity Index; ROS, Reactive oxygen species.

Scrotal isolation associated with dexamethasone treatment can also be used to relate the effects of testicular heating and the effect of stress on spermatogenesis in bulls, although similar results can be obtained in the types of morphological defects and in the temporal relationships of ascent and descent (155).

Mild testicular heat stress in bulls (8 h of scrotal isolation) has negative effects on the quality of frozen and thawed sperm (169). This was reported by Pérez-Crespo et al. (170) when studying the isolation of the scrotal neck in *B. taurus* bulls that showed an increase in scrotal-testicular temperature accompanied by an increase in the percentage of abnormalities between the head and the midpiece.

Some bull breeds, especially those crossbred between *B. taurus taurus x B. taurus indicus*, have no impact on semen quality during isolation from the scrotum. However, total isolation of the scrotum within 4 days is capable of reducing sperm production and quality in crossbred bulls (171).

In Nelore Bulls (*B. taurus indicus*), there may be a reduction in the amount of normal sperm and an increase in sperm with some type of head deficiency, mainly nuclear vacuoles and chromatin defects, during isolation from 14 to 21 days, after 5 days of total isolation of the bull scrotum (156).

On the other hand, in Brahman bulls (*B. taurus indicus*), submitted to 48 h of scrotal isolation, there was a tendency to decrease sperm motility. As a consequence, there was an increase in the rates of malformation of the head and cytoplasmic droplets, after 41 days of isolation (173).

In the case of mammals in the process of normal spermatogenesis, the testes need to be at room temperature, that is, in the range of 4–5°C lower than the body's core temperature (174). All this can be evidenced in most mammals, due to the location of the testes in the scrotum, therefore, outside the abdominal cavity (175).

The proportion of the vasculature and the lymphatic arrangement of the testes together with the superficial blood vessels of the scrotum seek to facilitate the removal of heat dissipation from the testes. The action of these neurons by the cold causes a reduction in the constriction of blood vessels while, on the other hand, the heat provides a vasodilation vasodilation. of these arterioles and, therefore, decreases or increases, respectively, the blood supply to the scrotum (176).

Beneath the surface of the scrotal skin is the tunica dartos, which plays an important role in thermoregulation. In this context, cutaneous muscle, specifically, a layer of smooth, thin muscle located under the tonic control of nerves corresponding to the lumbar sympathetic system that positions the scrotum towards the abdomen or further away from the abdomen in response to cold and environmental conditions (cold and hot, respectively) (113). As for the cremaster muscle, one of its contributions is to bring the testicles closer to the abdomen after contraction. Therefore, the striated nature of this muscle means that the muscle cannot sustain contraction for an extended period of time (95).

Other structures also favor the cooling of the testes, such as the vascular system. With regard to the testicular artery, one of its attributes is to transport warm blood from the body's core to the testicles. This artery is intertwined by a venous network known as the pampiniform plexus and this structure formed by the venous network plus the artery is called the testicular vascular cone (177).

**Figure 11:**
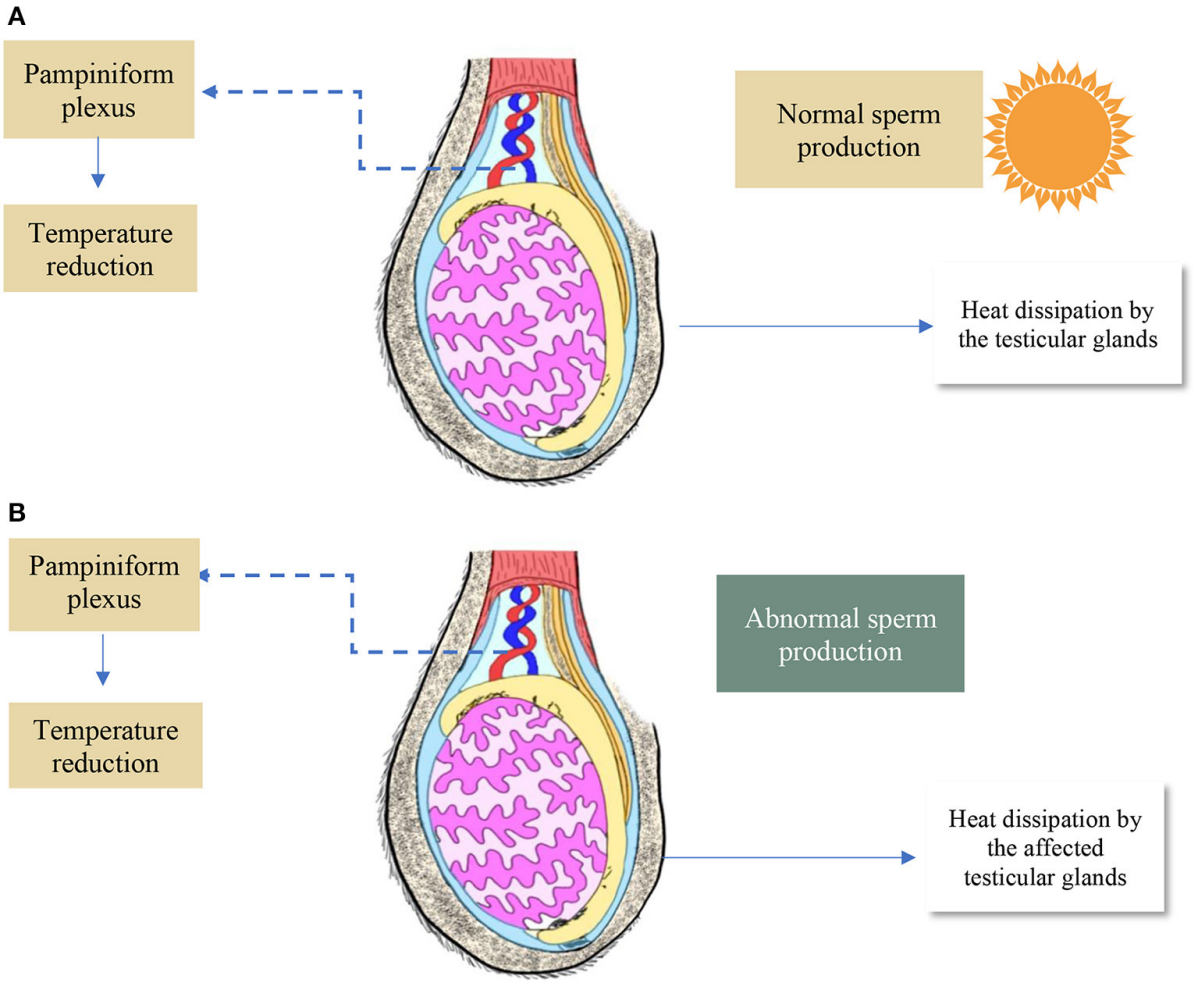
Bovine testis. **(A)** Morphological characteristics of the testis without heat stress. **(B)** Morphological characteristics of the testis under heat stress.

Because of this characteristic vasculature, a countercurrent blood circulation system may be evident in the testes. As a result of this circulation system, arterial blood entering the testes is cooled as venous blood is eliminated by the testes (Figures 11A, B).

According to Makker et al. (178), in bulls, sweat glands are another important factor. They also have attributes related to the control of testicular temperature, since the density of these glands is greater in the scrotal skin than in other regions of the body and for this reason—to a certain extent—bulls that have adequate scrotal circumference may have a greater ability to cope. with heat stress to some extent.

## Mechanisms of the effects of heat stress on spermatogenesis and semen quality

**Figure 12:**
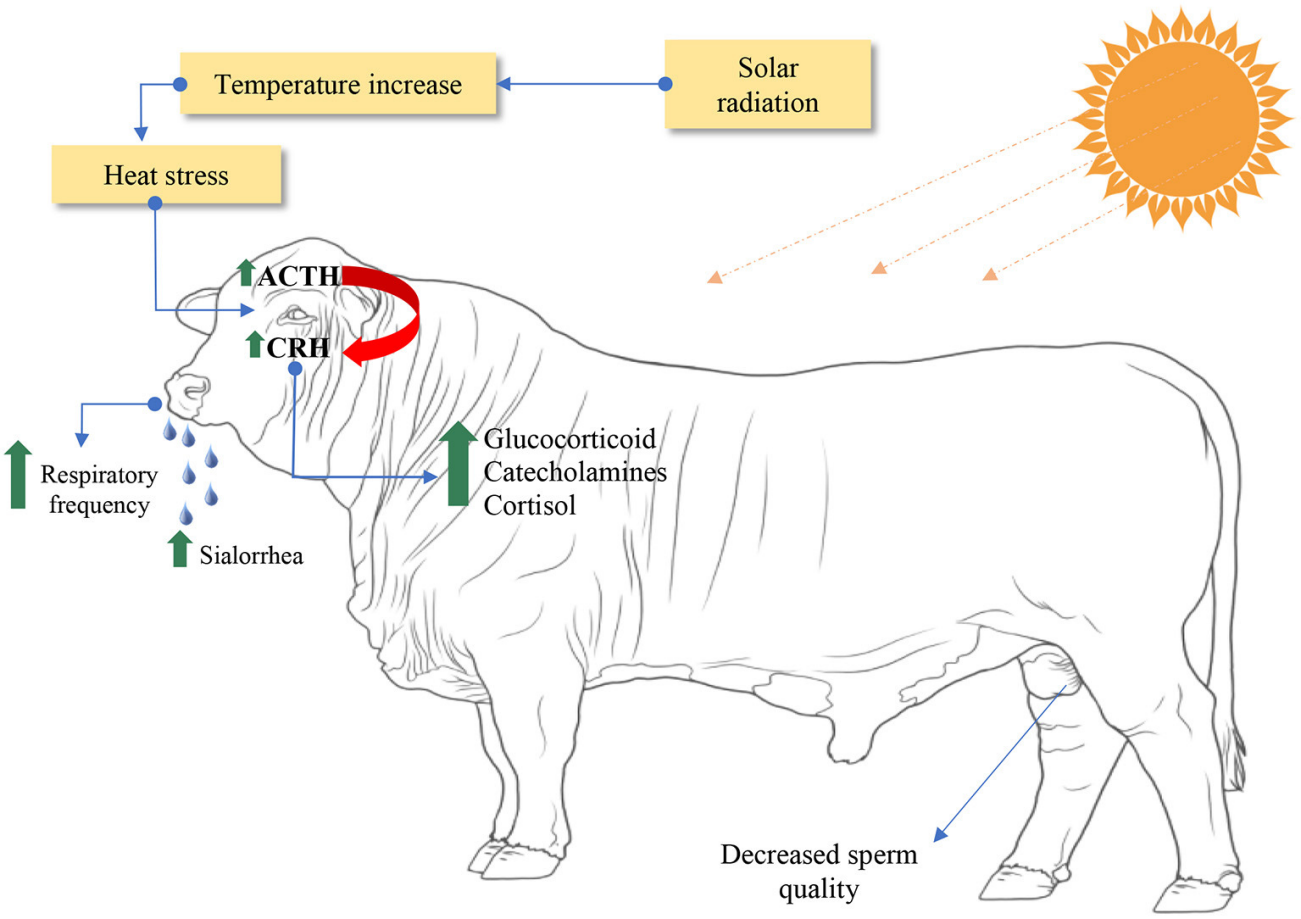
Illustration of the effects of heat stress on male bovine reproduction. ACTH, adrenocorticotropic hormone, or corticotropin; CRH, corticotropin releasing hormone. Adapted from Krishnan et al. (37) and Singh et al. (172).

The temperature resistance capacity is influenced by the duration of insolation (Figure 12), according to the degeneration of the germ cells that are induced (179). In this case, the exposure of male mice to a high temperature and humidity index compromises the sperm morphology and the integrity of the plasma membrane, disfavoring sperm mortality, and may even lead to infertility (163, 165, 170, 180).

The impacts caused by heat stress induce an increase in testicular temperature, evolving to sperm abnormalities. The appearance, proportion and severity of sperm abnormalities present in the ejaculate vary due to the intensity and duration of heat stress and the developmental stages of the affected germ cells (181).

It is noteworthy that the abnormalities are predominantly located in the sperm head, with emphasis on the presence of acrosomal defects, piriform heads, micro and macrocephalic heads (163). Sperm motility during 14–21 days can also be compromised by mild or short-term heat stress, resulting in aggravations in the spermatid and spermatocyte developmental stages (182, 183).

## Final considerations

Based on the information described in the literature on animal welfare and stress in reproductive aspects, it was possible to observe that the ability of thermal stress to negatively influence ovarian and testicular activity in cattle of different breeds, reared in different environmental and climatic conditions, is evident conditions, causing detrimental effects on spermatogenesis, sperm quality, folliculogenesis, ovulation, manifestation of estrus and embryo survival. In addition, other harmful consequences resulting from heat stress have also been reported, such as the increase in embryonic mortality rates that occurred in several countries, even in temperate climates. It is suggested to condition the animals in environments with shaded areas and the use of places that allow the animals to thermoregulate efficiently.

## Author contributions

Experiment design: WS, JS, and JBL-J. Experiment perform and writing—original draft: WS, JS, RC-J, ÉS, MS, RV, AS, CS, and JBL-J. Data curation: WS and JBL-J. All authors edited and approved the final manuscript.
